# Effect of the Tracheal Optical Properties on the Spectral Assessment of the Level of Its Blood Supply In Vivo

**DOI:** 10.3390/diagnostics15243170

**Published:** 2025-12-12

**Authors:** Anna Krivetskaya, Daniil Kustov, Tatiana Savelieva, Vladimir Parshin, Mikhail Ursov, Alexander Mariyko, Vladimir Levkin, Kirill Linkov, Sergey Kharnas, Mikhail Rusakov, Evgeny Sokolovich, Vladimir Makarov, Victor Loschenov

**Affiliations:** 1 Prokhorov General Physics Institute of the Russian Academy of Sciences, 119991 Moscow, Russia; 2 Institute of Engineering Physics for Biomedicine, National Research Nuclear University MEPhI, 115409 Moscow, Russia; 3 State Budgetary Healthcare Institution of the City of Moscow “S.S. Yudin City Clinical Hospital of the Moscow City Healthcare Department”, 117152 Moscow, Russia; 4 National Medical Research Center for Phthisiopulmonology and Infectious Diseases, 127473 Moscow, Russia; 5 Department of Faculty Surgery No. 1, I.M. Sechenov First Moscow State Medical University, 119435 Moscow, Russia; 6 Laboratory of Neurobiology and Tissue Engineering, Brain Science Institute, Research Center of Neurology, 125367 Moscow, Russia

**Keywords:** trachea, diffuse reflectance spectroscopy, blood supply, oxygen saturation, Monte Carlo method, complication prevention, diagnosis

## Abstract

**Background/Objectives:** This work is devoted to the presentation of the intraoperative method for assessing the blood supply to the tracheal tissues in order to minimize the risk of tissue ischemia and necrosis by early diagnosis of bloodstream disorders during surgery. The vascular network supplying the trachea is characterized by collateral blood circulation. However, after the surgical removal of a tracheal tumor, the created anastomosis may be untenable due to insufficient blood supply to the tissues. The consequence of such a disorder may require additional surgical interventions to restore the integrity of the trachea. Based on publicly available information, diffuse reflectance spectroscopy has not previously been used for blood supply assessment in tracheal surgeries. **Methods:** Light propagation in a four- or six-layer model of the tracheal wall (500–600 nm) was simulated using the Monte Carlo method; in the simulation, the layer thicknesses, levels of oxygen saturation (StO_2_) (0–100%), and blood filling (Hb% 0.5–2%) were varied. Intraoperative measurements using diffuse reflectance spectroscopy were performed in 12 patients at three stages of the operation. **Results:** The simulation showed that when the fiber is placed from the adventitial side, the differences in the diffuse scattered signal with changes in perichondrium saturation are 2.6 ± 1.7%, whereas when placed from the mucosa side, the changes are less than 1%, which means that deeper layers make a greater contribution to the signal with adventitial access. When testing the StO_2_ estimation algorithm, the simulation and experiment agree: the measured StO_2_ was 56 ± 7%, which is close to the specified level in the simulation. Clinical measurements demonstrated the possibility of recording saturation changes at the stages before and after the anastomosis. **Conclusions:** According to the results of this study, saturation estimation by diffuse reflectance spectroscopy shows the prospect for assessing the state of tracheal tissues by the level of their blood supply in clinical conditions in real time.

## 1. Introduction

The main risk factor for anastomotic failure after tracheal surgery is the degree of tension of the sutured tissues [1,2]. Tissue tension affects the level of local blood supply: with an increase in the degree of tension, blood supply to the anastomotic area decreases [3]. In addition, the presence of ischemia also affects the incidence of serious postoperative complications [4]. Accordingly, the level of blood supply, including local oxygen saturation, affects the risk of anastomotic leakage [5,6,7].

There are several conditions of biological tissues that may affect the treatment outcome, including the blood supply level (tissue saturation, blood filling) and the structural state (optical properties). Currently, the assessment of blood supply parameters such as the blood filling and oxygenation of tracheal tissues during surgery is not generally accepted. Intraoperative assessment of the oxygen saturation level of biological tissues during a surgical procedure can influence the outcome of the operation and reduce the risk of postoperative complications associated with insufficient blood supply and hemoglobin oxygenation [8,9]. Blood filling and hemoglobin oxygenation are key factors in determining the adequacy of blood supply as a result of the performed surgery. Insufficient blood supply leads to anastomotic failure, inflammation of the operated tissues, a long postoperative period, and an increased risk of reoperation [10,11]. Intraoperative assessment of the state of tracheal tissues can be implemented using various methods, including those based on a radiation interaction study in the optical range with tissues.

There are several alternative and complementary approaches to assessing tissue perfusion and oxygenation. One of the methods that allows obtaining information about the condition of biological tissues is optical coherence tomography [12,13,14]. This technique enables the visualization of tracheal tissues, endoscopic investigation, and, accordingly, detecting the presence of any lesions, but it does not provide quantitative data about the condition of the tissues. This determines the possibility of differences in the interpretation of the diagnostics results. An extension of this method is optical coherence angiography (OCA), which allows for the assessment of blood flow by examining the same area several times and processing signal changes over time. However, diagnostics using this technique take a significant amount of time. For example, if a blood vessel is bleeding, OCA may not provide a timely image to medical personnel, and the delay in imaging may lead to retentions in intervention and may affect the results of the surgery. In addition, obtaining quantitative information in this case is also impossible [15]. The method developed in the work [16] is intended to eliminate these disadvantages. Near infrared (NIR) spectroscopy is used for point-by-point quantitative measurement of StO_2_. The main difference between this method and the one presented in the current work is the analyzed spectral region. The rationale for choosing the spectral region analyzed in this research is given in the Materials and Methods section. Further, the condition of the tracheal wall can be assessed using fluorescence diagnostics [17], including intraoperative indocyanine green fluorescence angiography (ICG-FA), which provides real visualization of the blood flow [18]. The disadvantage of this technique in the context of blood supply studies is the necessity to introduce a photosensitizer into the patient’s body. If the fluorescent agent is not introduced properly, or there is a delay in its consumption by the tissue, this may result in unclear or inaccurate images and may interfere with the detection of important vascular structures and tissue perfusion during surgery. In addition, the photosensitizer administration places additional stress on the patient. Hyperspectral imaging (HSI) [19] and laser speckle contrast imaging (LSCI) [20] provide non-contact maps of the StO_2_ and relative blood flow, respectively. HSI is based on recording an image of a single field of view in several spectral ranges using a set of narrow-band filters or using several narrow-band light sources [21]. However, this method is time-consuming and can be inconvenient during surgery due to both shift artifacts and significant variations in the height of the surface relative to the receiver. LSCI allows the assessment of the state of biological tissues by determining their degree of blood supply [22,23,24,25]. However, despite the ongoing work in this area [26], this technique also does not provide quantitative data and has low comparability between patients [27]. Other possible methods for evaluating the tracheal tissue condition include autofluorescence [28,29] and narrow-band imaging. The disadvantage of autofluorescence is a weak signal intensity and low specificity [30]. Diagnostics with the narrow-band imaging method take a long time. Moreover, experience is required for the correct interpretation of the results, since this technique does not provide quantitative data [31]. Photoacoustic imaging (PAI) provides a spectral assessment of oxygenation with greater penetration depth [32,33]. However, in our work, a large depth of research is not required; therefore, the use of an ultrasonic source in addition to optical radiation is not justified. Higher-resolution optical techniques such as in vivo and confocal microscopy provide visualization at the cellular level. However, these techniques only provide insight into very superficial layers and small areas of the examined organ. The optical properties of biological tissues limit the depth of investigation to a few hundred micrometers. Another technique that provides information on the tissue state is Raman spectroscopy [34]. This method allows differentiating between malignant and non-pathological tissues. Each method considered has its own limitations in terms of the ability to provide quantitative data, spatial/temporal resolution, and the need for contrast; so, a combined approach is optimal depending on the clinical task.

In this work, the developed method based on diffuse reflectance spectroscopy (DRS) was proposed to determine the condition of tracheal tissue by assessing the degree of blood flow. It has been previously shown that optical spectral methods allow the quantitative assessment of the oxygenation level in biological tissues in clinical [35,36,37,38] and laboratory conditions [39]. Based on the publicly available information, this technique has not previously been used in tracheal surgeries. Our work proposes carrying out measurements from both the mucosal membrane and the adventitial layer to assess the maximum possible volume of the organ wall. Other advantages of the considered method include non-invasiveness and the absence of the need to excise a tissue sample or introduce an additional substance into the patient’s body. The purpose of this work is the intraoperative assessment of the tracheal tissues’ blood supply based on the measured values of their oxygen saturation and blood filling and a comparison of the experimental data with the results obtained during simulation of the light propagation in tracheal tissues using the Monte Carlo method.

## 2. Materials and Methods

### 2.1. Tracheal Layers

**Figure 1:**
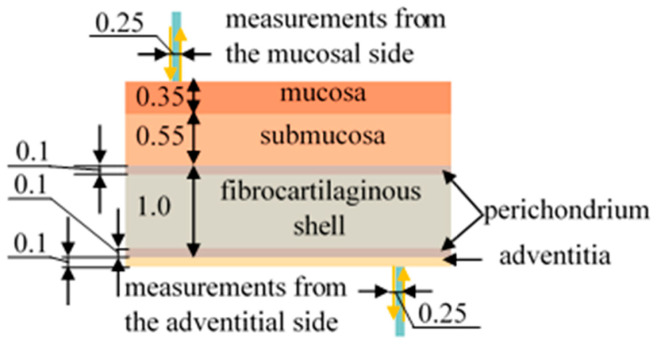
Schematic representation of the tracheal layers and the process of measuring diffuse reflectance spectra using optical fiber. All dimensions are in the units of mm.

The structure of the tracheal wall differs for its posterior part as well as for its anterior and lateral sections. The anterior and lateral walls of the trachea, starting from the lumen of this organ, consist of mucosal, submucosal, fibrocartilaginous, and adventitial layers, while at the posterior wall, the cartilaginous membrane is replaced by a muscular layer, and the adventitia passes into a membranous wall [40]. Figure 1 shows a demonstration of the layers of the organ under study.

Since this work is devoted to the assessment of the blood supply to the tracheal tis-sues, it is important to consider which layers of this organ contain blood vessels. According to the literature data [41,42], vessels are located in each of the layers of the trachea; however, in the fibrocartilaginous membrane they are not located throughout the entire volume, but only in the perichondrium, which is placed along its edges on both sides.

### 2.2. Diffuse Reflectance Spectroscopy

**Figure 2:**
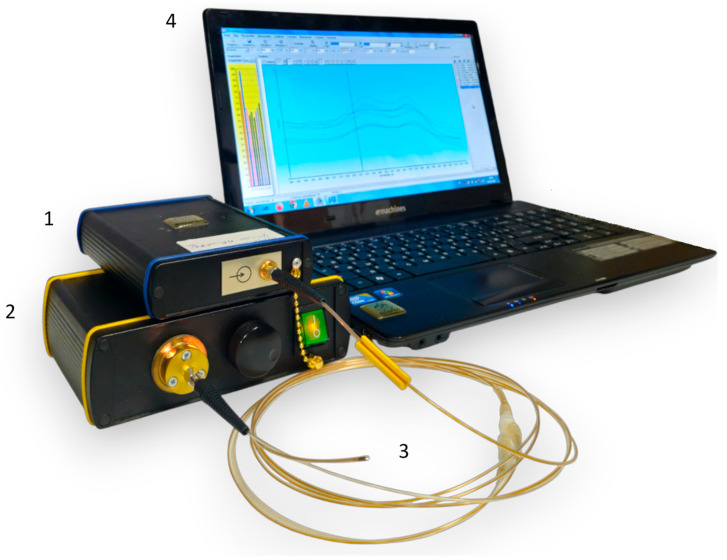
Photograph of the equipment for spectral measurements. (1) A “LESA-01-BIOSPEC” spectrometer, (2) a broadband light source, (3) a Y-shaped optical probe, and (4) a laptop with the interface of the “Uno Momento” program on the screen.

**Figure 3:**
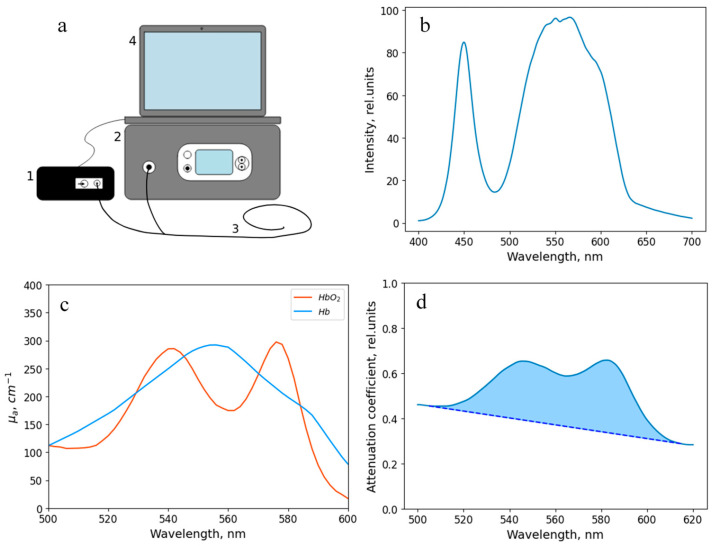
(**a**) Scheme of the experimental setup. The equipment includes (1) a “LESA-01-BIOSPEC” spectrometer, (2) a broadband radiation source, (3) a Y-shaped optical fiber, and (4) a computer with the “Uno Momento” program. (**b**) Spectral characteristics of the broadband radiation source. (**c**) Absorption coefficient of hemoglobin in oxygenated and reduced forms. (**d**) An example of a spectrum recorded during the study. The blue region shows the area analyzed to assess the level of blood filling.

For intraoperative assessment of the tracheal tissue condition based on the blood circulation level, the developed method based on diffuse reflectance spectroscopy was used. This technique allows measuring the level of oxygen saturation of the microcirculatory bloodstream of the tracheal tissues (StO_2_), as well as the degree of blood filling by processing the recorded spectra and, accordingly, provides a quantitative characteristic for an objective assessment of the condition of the tracheal wall. The equipment for spectral measurements is presented in Figure 2. It includes a “LESA-01-BIOSPEC” spectrometer, a broadband light source based on LEDs, an optical probe, and a PC with the “UnoMomento” program (Figure 3a). All components of the set-up, excluding the PC, were developed in the BIOSPEC company (Moscow, Russia), which is located in Russia.

The output power of the source was 1–5 mW. The spectral characteristic of the broadband radiation source is shown in Figure 3b.

**Figure 4:**
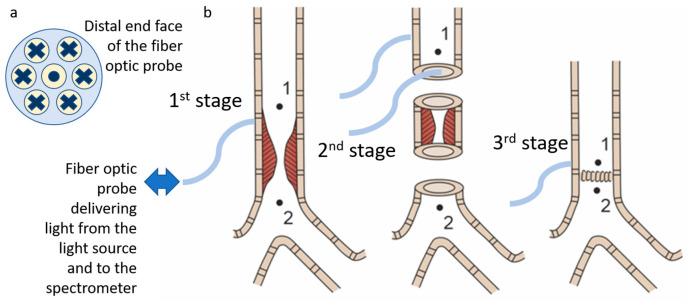
(**a**) Scheme of the fiber placement inside the distal end of the optical probe. The dot and crossings indicate the direction of the light transport. (**b**) Scheme of the surgical stages with designation of the points at which spectrometric analysis of the blood supply level was performed.

The optical probe, which was used to transmit the radiation, has a Y-shape. One end of the probe is connected to the spectrometer, the other one is connected to the light source, and the distal end is brought to the object under study. The total diameter of the optical probe is 1.7 mm, and the diameter of each of the 7 optical fibers (one for illumination and six for signal transfer to the spectrometer (Figure 4a)) located inside is 250 μm. The layout of the optical fibers inside the probe and the extensive algorithm for determining the StO_2_ level are presented in a previous work [43]. The range studied to assess the level of tissue oxygen saturation lies in the wavelength region of 500–600 nm. The spectral resolution of the spectrometer was 2 nm. The dependence of the absorption coefficient on the wavelength in this spectral range is shown in Figure 3c. The sterilization of the optical probe was carried out along with the endoscopic equipment.

The use of the analyzed spectral region in the range of 500–600 nm in this study is governed by the fact that hemoglobin is one of the main absorbers of biological tissues in this region, as well as by the presence of differences in the shapes of the absorption spectrum of oxygenated and deoxygenated hemoglobin (Figure 3c). A more detailed justification for the choice of the analyzed spectral range is given in the work [38].

Signal attenuation in the wavelength range of 500–600 nm is mainly conditioned by the absorption of light by hemoglobin in oxygenated and reduced forms, which makes it possible to calculate the reference values of the attenuation coefficient:

(1) A = c_0_ + c_1_λ + c_2_λ_2_ + <L>·{c_Hb_·ε_Hb_(λ) + c_HbO2_·ε_HbO2_(λ)} · ln(10),

where λ is the wavelength, <L> is the mean pathlength of photons in tissue between the lighting and receiving fibers, c_Hb_ is the reduced hemoglobin concentration, c_HbO2_ is the oxygenated hemoglobin concentration, ε_Hb_ is the molar extinction coefficient of reduced hemoglobin, ε_HbO2_ is the molar extinction coefficient of oxygenated hemoglobin, and c_0,_ c_1_, and c_0_ are fitting coefficients.

The experimental values of the attenuation coefficient were determined by calculating the logarithm of the ratio of the spectrum from barium sulfate, which is the reference reflector (I_0_), and the signal recorded from the object under study (I):

(2) A = ln(I_0_/I).

After that, by finding the minimum value of the discrepancy, the concentrations of the oxygenated and reduced forms of hemoglobin were determined, and the saturation was calculated using the formula:

(3) StO_2_ = c_HbO2_/(c_HbO2_ + c_Hb_).

In addition to the determination of the oxygen saturation, the method presented in this paper allows for a comparative analysis of the blood filling level of the tissues under study based on the obtained diffuse scattering spectra. This parameter is determined by the area between the recorded spectrum in the spectral region under study and the tangent to it (Figure 3d).

To perform registration of diffuse reflectance spectra, the distal end of the optical probe was placed in soft contact with the tracheal tissues. The spectra were registered at the three stages during surgery (Figure 4b). The first stage included measurements on the intact tracheal tissues. In this case, signal detection was performed only from the adventitial side. The second phase was after the removal of the abnormal part of the trachea: the measurements were taken both from the external and intraluminal side of the trachea. The third stage was after the anastomosis application: measurements were carried out from the adventitial side. At each stage, the measurements were taken cranially (point 1 in Figure 4b) and caudally (point 2 in Figure 4b) to the area of interest.

### 2.3. Monte Carlo Simulation

The Monte Carlo method was used to simulate the light propagation in tracheal tis-sues. Applicable to the light propagation in tissue, this method consists of the repeating calculation of the photon trajectory in a medium, based on the specified parameters of the medium, including its optical properties and thickness. The rules for photon transfer are determined using probability distributions that describe the step size of a photon movement between the sites of its interaction with matter and the angles of deviation of the photon trajectory when a scattering event occurs [44].

The light beam shape was set to match the propagation of radiation from an optical fiber with specified parameters. The numerical aperture of the illuminating and detecting optical fibers was set to 0.37, and the diameter was set to 0.25 mm. When simulating the placement of the illuminating and receiving fibers on one side of the biological tissue surface, the distance between the illuminating and receiving fibers was set to 0.25 mm, in accordance with the setup configuration used for the clinical recording of diffuse scattering spectra. When the fibers were located on different sides of the tracheal wall, the source and receiver were installed opposite each other. The number of simulated photons was 10^7^ for each calculation run. The Monte Carlo statistical uncertainty scales as 1/√N (≈0.0316% for N = 10^7^), which yields negligible photon-count noise compared with uncertainties in optical parameters and, therefore, provides stable fluence/reflectance profiles for subsequent spectral unmixing and StO_2_ extraction.

To perform the calculations, the tracheal wall was represented in the form of four (mucosa, submucosa, fibrocartilage, and adventitia) or six (with fibrocartilage divided into three parts: upper pericondrium, central part, and lower pericondrium) layers with given optical properties and thickness. The parameters of each layer, for which the modeling was carried out, such as the thickness, scattering and absorption coefficients, as well as the anisotropy factor, are presented in Table 1 [45,46,47,48,49,50,51]. The refractive index for all layers was assumed to be 1.37 [52,53]. The angular propagation of light in biological tissues was performed using the Henyey–Greenstein phase function [54,55].

In order to verify the feasibility of using the proposed method in clinical practice, the light propagation in tracheal tissues was simulated with the fiber positioned on the mucosal and adventitial side with different degrees of hemoglobin oxygen saturation in the perichondrium. The StO_2_ values of the other layers remained unchanged. This configuration allows determining whether the results of measurements using diffuse reflectance spectroscopy are affected by the saturation level of the fibrocartilaginous membrane, or whether this method evaluates the state of only the superficial layers. The variation in the StO_2_ level was achieved by changing the absorption coefficient of the perichondrium in accordance with the properties of the oxygenated and reduced forms of hemoglobin, considering the percentage of hemoglobin in the tissues to be equal to 1%.

The modeling was also performed with different thicknesses of each of the tracheal layers. When changing the thickness of one of the layers, the thicknesses of the remaining layers remained unchanged: 0.35 mm for the mucosa, 0.55 mm for the submucosa, 1 mm for the fibrocartilaginous membrane, 0.1 mm for the adventitia. The varied values were 0.15, 0.25, 0.35, 0.45, and 0.55 mm for the mucosa, 0.35, 0.45, 0.55, 0.65, and 0.75 mm for the submucosa, 0.7, 0.85, 1.0, 1.15, and 1.3 mm for the fibrocartilaginous membrane, and 0.05, 0.075, 0.1, 0.15, and 0.2 mm for the adventitia. These ranges correspond to those possible in real cases. The parameters of the layers (their optical properties) corresponded to the values given above (Table 1). The modeling was carried out with the illumination fiber positioned on the mucous membrane side and on the adventitial membrane side.

To study the effect of different levels of tracheal tissue blood supply on the detected signal, the simulation was performed by varying the StO_2_ values and the blood filling level. To simulate a change in the oxygen saturation level, the absorption coefficient values were changed in accordance with the properties of the oxygenated and reduced forms of hemoglobin. The hemoglobin content in the tissues was considered to be 1%. Simulation was performed at an StO_2_ level of 0, 25, 50, 65, 80, and 100%. The blood filling level was varied by changing the percentage of hemoglobin in the tissues, namely at a level of 0.5, 1, and 2%. In this case, the tissue oxygen saturation level was fixed at 50%.

### 2.4. Patients

Clinical studies were conducted at the First Moscow State Medical University named after I.M. Sechenov and the National Medical Research Center for Phthisiopulmonology and Infectious Diseases. The study involved 12 patients who underwent circular resection of the trachea. Eight patients were diagnosed with stenosis, and four were treated due to the presence of neoplasms. Malignant tumors were presented by the squamous cell keratinizing carcinoma of the cervical and upper thoracic tracheal sections. During the clinical study, the saturation level was determined intraoperatively at three stages: before the trachea was crossed, after part of the organ was removed, and after the anastomosis was formed.

## 3. Results

### 3.1. Monte Carlo Simulation of Light Propagation in Tracheal Layers

Modeling of light propagation at wavelengths in the visible range allowed us to study the processes of light interaction with tracheal tissues.

**Figure 5:**
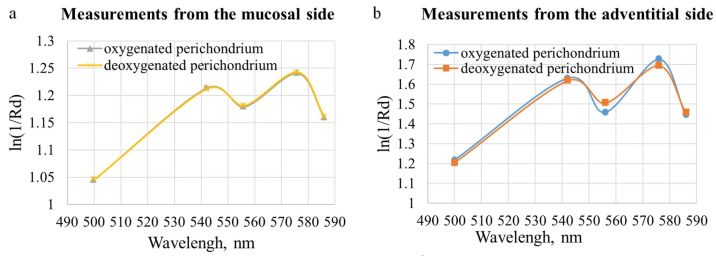
Modeled dependences of the absorption magnitude of the tracheal wall on the wavelength with a change in the degree of saturation of the perichondrium with oxygen when the fiber was placed (**a**) from the side of the mucosal layer and (**b**) from the side of the adventitia.

When modeling the propagation of light in tracheal tissues using six-layer prototype with varying degrees of hemoglobin oxygen saturation in the perichondrium with unchanged StO_2_ in the other layers, the difference in the diffuse reflectance from the adventitia side is 2.6 ± 1.7%, while the difference from the mucosa side is less than one percent. These results are shown in Figure 5. It is evident that during simulation of the fiber position on the mucosal side, the differences in the dependencies for oxygenated and deoxygenated perichondrium are practically unnoticeable. While during simulation of the fiber position on the adventitial side, the graphs visually differ with changes in perichondrium saturation. This is explained by the ratio of the layer thicknesses and the probing depth at a distance of 250 μm between the illuminating and receiving fibers (Figure 1). Therefore, to assess the condition of the fibrocartilaginous membrane, saturation measurements should be taken from the adventitia. Measurements from the lumen of the trachea provide information on the condition of the mucosal and submucosal layers.

**Figure 6:**
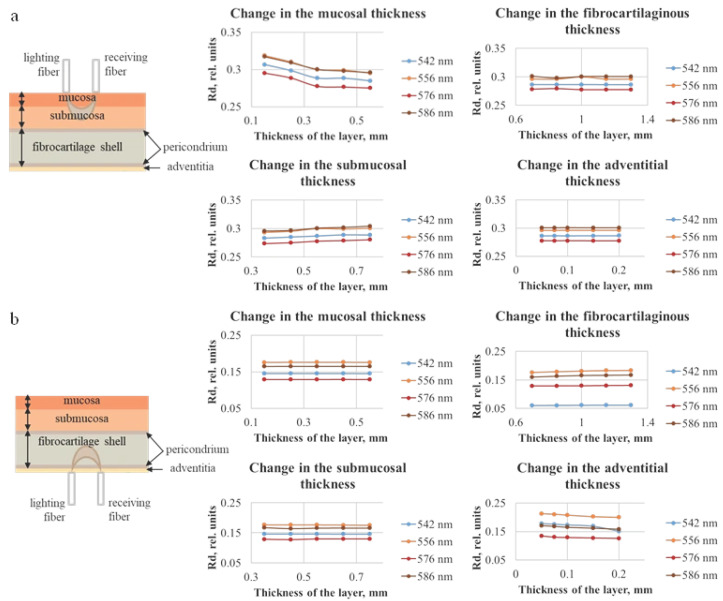
Dependences of diffuse reflection of the tracheal wall for different thicknesses of each of the four tracheal layers, which were obtained using the Monte Carlo simulation method, with the illumination fiber placed (**a**) on the side of the mucosal layer and (**b**) on the side of the adventitia.

**Figure 7:**
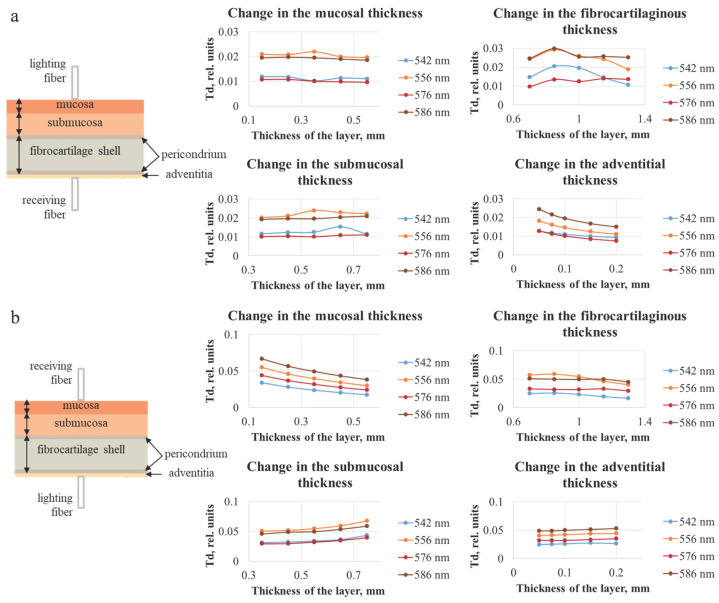
Dependences of the diffuse transmittance of the tracheal wall for different thicknesses of each of the four layers of the trachea, obtained using the Monte Carlo simulation method, with the illumination fiber placed (**a**) on the side of the mucosal layer and (**b**) on the side of the adventitia. The plots illustrate the stronger influence of the layer adjacent to the detector on the detected transmission signal.

The dependences of diffuse reflectance and transmittance of the tracheal wall were also obtained for different thicknesses of each of its layers. The distance between the lighting and receiving fibers was set equal to 0.25 mm, which corresponds to the considered distance between the fibers in the optical probe used for clinical spectral measurements. The trachea was represented as six-layer structure. The obtained dependences are shown in Figure 6 and Figure 7.

Figure 6 shows the diffuse reflection (Rd) values, which for 556 and 586 nm are higher than for 542 and 576 nm. This corresponds to the absorption properties of the oxygenated form of hemoglobin. The effect on the magnitude of the diffusely reflected signal is observed only when changing the thickness of the two layers closest to the fiber, which indicates that to assess the state of the tracheal wall over the entire thickness, measurements should be taken from both the adventitia and the mucosa.

Figure 7 indicates that, with the increase in the layer thickness, the transmission either remains approximately at the same level or decreases, which can be explained by the fact that, with a larger thickness, the detected radiation must travel a longer distance, and a larger number of photons will be absorbed and scattered. In addition, analogously to the data for diffuse reflection, the results of modeling the diffuse transmission (Td) are consistent with the absorption properties of oxygenated hemoglobin. It can be seen that the layer on the side of the receiving fiber has the largest influence on the transmission signal. In Figure 7a the adventitial layer could be neglected due to its diminutive thickness (less than 0.2 mm); hence, the receiving fiber is located on the fibrocartilaginous side. In Figure 7b, this layer is the farthest from the receiver, and the highest contribution to the signal is conditioned by the change in the mucosal layer. The thickness of the fibrocartilaginous layer varies over a wider range than the thickness of the mucosa; for this reason, there is a steeper dependence of the Td signal on the thickness in Figure 7a.

**Figure 8:**
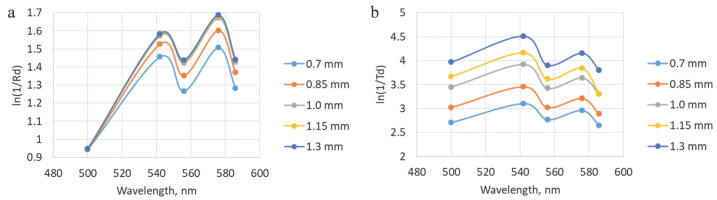
Illustration of dependencies obtained after processing the modeling results, which correspond to the experimental spectra. (**a**) Data for different thicknesses of the fibrocartilaginous membrane with modeling of the fibers placement from the adventitial side. (**b**) Data for different thicknesses of the fibrocartilaginous membrane with modeling of the placement of the lighting fiber from the mucosal side.

To make the comparison between the modeling and experimental data more visual, the coefficients of diffuse reflection and transmission were recalculated into the logarithm of the ratio of one to the specified parameters. In this form, the obtained dependencies correspond to the spectra obtained during the measurement process. Figure 8 shows examples of the results of material processing. It is evident that the blood filling level increases with the increasing layer thickness, which is tenable, given the homogeneity of the specified properties.

**Figure 9:**
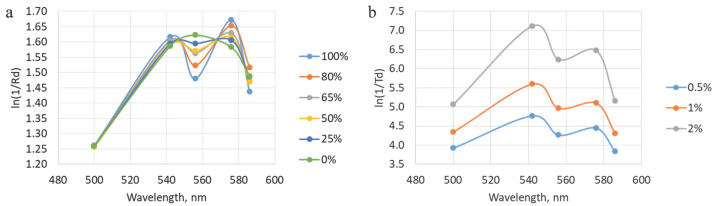
(**a**) Dependencies obtained during modeling with different levels of tracheal wall oxygen saturation with the fibers located on the mucosal side. (**b**) A representation of data acquired during processing of the modeling results with variations in blood filling values. The shown graphs correspond to the location of the illuminating fiber on the adventitial layer side.

The results of the analysis with different oxygen saturation levels when converted to the absorption coefficient (ln(1/Rd)) correspond to the expected ones. At StO_2_ equal to 100%, two peaks are visualized at wavelengths of 542 and 576 nm. While at 0% level, only one peak is observed. The diffuse reflectance values obtained during the simulation with a change in blood filling levels were converted to an attenuation coefficient, after which the StO_2_ degrees were assessed. The extracted saturation level was 56 ± 7%, which corresponds to the value specified in the simulation (50%). In this case, the four-layer model of the trachea was used. The results of the analysis at different saturation and blood filling levels are presented in Figure 9.

### 3.2. Clinical Investigation

**Figure 10:**
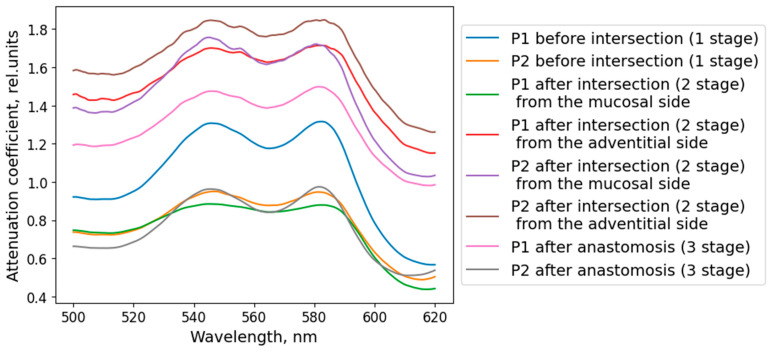
Diffuse scattering spectra averaged over the studied localization, obtained during the clinical study of the blood supply to the tracheal tissue of the patient.

The oxygen saturation level of the microcirculatory bed of the tracheal tissues and the degree of blood filling were measured at several stages of the surgical intervention: before the trachea was transected, after the damaged part of the trachea was removed (at this stage the measurements were taken from the mucosa and from the adventitia), and after the anastomosis was created (Figure 4b). To increase the credibility of the results, at least five spectra were recorded in each area of measurement. When processing the results of the clinical measurements, the spectra related to one area of study were averaged for each of the measurement stages. Examples of diffuse scattering spectra obtained after this stage of processing are shown in Figure 10.

**Figure 11:**
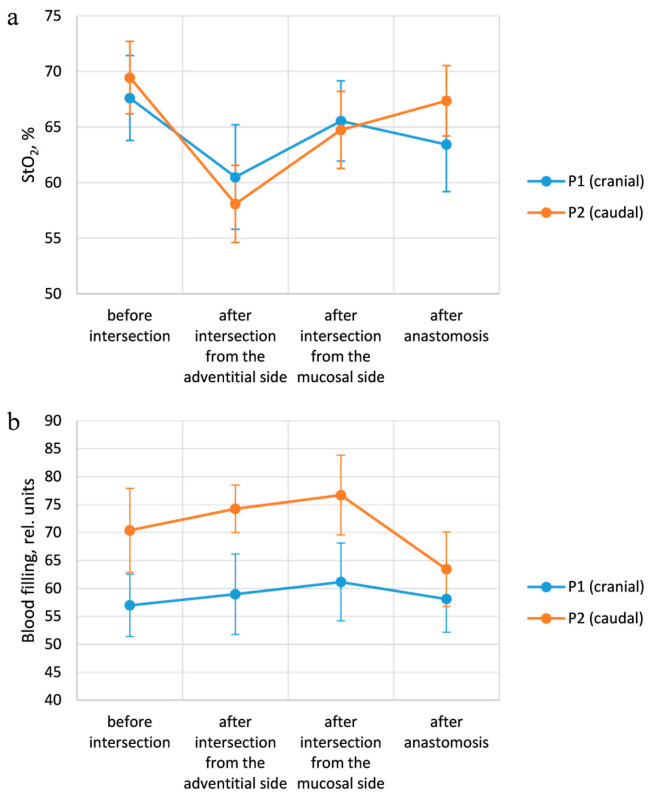
Results of intraoperative measurements. (**a**) Changes in StO_2_ values, depending on the measurement points at each stage of the operation. (**b**) Dependence of the blood filling level on the stage of the surgery for each examined area.

Figure 11a shows the averaged intraoperative saturation values for all patients at each stage. The error bars represent the standard deviation. As can be seen from the graph, the observed saturation level after the transection was lower than at the initial measurement. The saturation values after the anastomosis were restored to the level that was before the transection of the trachea, indicating an acceptable level of blood supply to the sutured tissues. These results correspond to the predominant absence of postoperative complications; anastomotic failure was observed only in one case and was caused by mechanical damage.

The variation in the blood filling level of the tracheal tissues depending on the stage of the operation is presented in Figure 11b. The indicated values correspond to the results averaged over all patients. After the organ was crossed, the blood filling values increased, which is associated with the blood inflow to the surgical intervention area. Moreover, at the second stage of the operation, the blood filling level measured from the adventitial side is lower than with the fiber placement from the lumen side, which corresponds to the more developed vascular network of the mucosal layer. After the anastomosis is imposed, the blood filling level in the cranial region returns to the values at the first stage of measurements, while in the caudal region, the parameter in question is lower than at the previous stages. These results indicate a partial restoration of blood supply; however, more time is required for complete restoration. In addition, the blood supply level in the caudal region at all stages of measurement is higher than in the cranial one. This can be explained by the location of the vessels providing blood supply to the trachea. During the transection of the organ in consideration, the vessels which supply the part of the trachea cranial to the incision area are damaged, while the blood supply to the caudal region remains largely unchanged.

### 3.3. Correlation of Clinical Measurements with Modeling Results

According to the results derived during the intraoperative assessment of tracheal tissue oxygen saturation, it is apparent that at the second stage of measurements, the values obtained when the fiber was placed on the adventitial side are lower than when the fiber was placed on the mucosal side. Based on the modeling results, more precise information on the state of the fibrocartilaginous membrane can be obtained by investigation from the adventitia side. Accordingly, it can be concluded that the lower StO_2_ values for the fiber placement on the adventitial side during clinical measurements are conditioned by the reduced blood supply to the fibrocartilaginous membrane compared to the mucosal and submucosal layers.

In this work, it has been shown that a combination of Monte Carlo simulation and diffuse reflectance spectroscopy allows recording and quantifying the blood supply parameters of different layers of the tracheal wall under surgical conditions. The most important observations are the agreement between the extracted StO_2_ values and the simulated scenarios and the pronounced sensitivity of the recordings with the adventitial probe placement (the contribution of the fibrocartilaginous layer to the spectral signal was clearly higher in this case than with the mucosal approach). This indicates that the source-detector geometry and the contact point determine which tissue layers will dominate the measurement: the adventitial approach better reflects the state of the deep structures (fibrocartilaginous layer), while the mucosal approach provides information mainly on the intraluminal microcirculation.

The obtained agreement between the simulation and the experimental data increases the confidence in the StO_2_ extraction algorithm but does not eliminate the need for further validation. The models serve as a qualitative guide and help explain the directionality of signals, but the absolute values depend on a number of assumptions (optical properties of layers, homogeneity, hemoglobin content, etc.). Therefore, the interpretation of results should be based on both the model and independent clinical references in subsequent studies.

**Figure 12:**
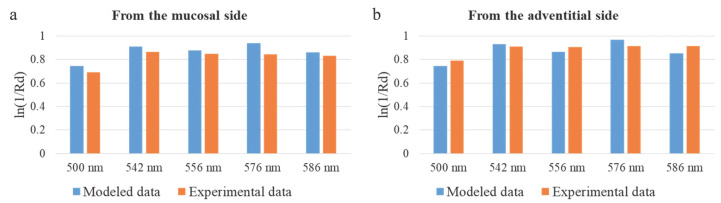
Comparison of the attenuation coefficient in diffuse reflectance geometry for modeled and experimental data. (**a**) Results for the fiber placement from the mucosal side. (**b**) Values for the fiber placement from the adventitial side.

The agreement between the simulation and clinical measurements is further substantiated by comparing the attenuation profiles. The Monte Carlo model identified the perichondrium, due to its high blood content, as a layer with a high absorption coefficient. Clinically, this was reflected in the consistently higher measured attenuation coefficient (−ln(Rd)) when probing from the adventitial side compared to the mucosal side. This correlation, visually supported by Figure 12, indicates a higher contribution from the perfused perichondrium to the signal when accessed from the adventitia, exactly as predicted by the model. Therefore, the lower StO_2_ values recorded from the adventitial side (Figure 11a) are not an artifact but a direct consequence of the method’s sensitivity to the deep fibrocartilaginous layer, the oxygenation of which is critically important for anastomotic healing.

## 4. Discussion

### 4.1. Peculiarities of the Developed Method

Comparing our approach with the existing clinical and research methods, it should be noted that diffuse reflectance spectroscopy occupies an intermediate position in a number of criteria: the method is non-contrast and relatively easy to use (in the form of a compact fiber probe), but it provides point information and, therefore, is limited in area coverage. This makes it a good complement to the already used intraoperative techniques, rather than a full-fledged replacement. In clinical practice, the most common and evidence-based method for assessing intraoperative perfusion remains indocyanine green fluorescence angiography, which, according to the results of meta-analyses, is associated with a decrease in the incidence of anastomotic leaks in abdominal (in particular, colorectal) surgery; however, this method requires the introduction of a contrast agent and provides mainly a superficial picture of blood flow [18]. Technical optimization of the method may involve the introduction of new up-conversion materials to enhance the near-infrared sensitivity and increase the depth of the detected signal; such technologies have already shown high NIR-to-NIR conversion efficiency and can be used to improve the signal-to-noise ratio in tissue spectral measurements [56].

Non-contrast wide-field approaches such as hyperspectral imaging and laser speck-le contrast imaging provide spatial maps of parameters (StO_2_ or relative blood flow maps) and are suitable for the real-time imaging of large tissue areas; these methods are being actively developed and show promising clinical applications but still require the standardization of data processing and calibration. HSI is particularly useful, where the spectral content of reflected light and field-wide mapping of oxygenation are important, while LSCI provides fast relative blood flow maps with very good temporal resolution [57]. Photoacoustic imaging is a promising direction that can overcome one of the main limitations of optical methods—penetration depth. By combining optical contrast (Hb/HbO_2_ sensitivity) and acoustic detection, PAI provides oxygenation information at greater depths than purely optical methods and is thus potentially better suited for assessing the deep layers of the tracheal wall; however, the clinical translation of PAI is still in an active development stage and requires solving engineering and regulatory challenges [58].

The clinical practical value of fiber spectroscopy lies in its ability to complement existing methods: it can serve as a targeted tool for assessing suspicious areas around the anastomosis, helping the surgeon decide on additional measures (change in resection level, additional tissue mobilization, or vascular reinforcement). The limitations of our study are typical for pilot studies: small sample size, simplifications in modeling (fixed optical parameters, layer homogeneity), spotty nature of measurements and potential artifacts (probe contact, blood/fibrin presence, movement). An additional promising possibility for expanding the method may be the use of nanomaterials that are spectrally sensitive to inflammatory processes, which, when accumulated in the resection zone, are capable of providing enhanced optical contrast and simultaneously serving as a platform for therapeutic interventions [59].

### 4.2. Collation with the Other Organs of the Respiratory System

Intraoperative assessment of tracheal tissues typically involves only direct visualization by the surgeon or via bronchoscopy [4,60]. A thorough literature search confirms that there are no publicly available works dedicated specifically to the application of diffuse reflectance spectroscopy (DRS) for assessing tracheal tissue blood supply during surgical operations. To contextualize our approach, it is, therefore, appropriate to consider the application of DRS and related optical techniques to other organs of the respiratory system, which demonstrates the general feasibility of optical methods in this anatomical domain.

Research into DRS for lung and bronchial tissues is well-established, primarily focusing on diagnostic differentiation. Studies on resected lung tissue [61] and in vivo during bronchoscopy [62,63,64] have demonstrated that DRS can distinguish malignant from non-malignant tissue with high sensitivity and specificity. This body of work confirms the utility of DRS for real-time tissue characterization in the respiratory tract. Furthermore, a combined approach using autofluorescence and DRS has shown even higher accuracy for lung cancer detection in ex vivo samples [65], highlighting the potential of multi-modal optical systems.

Beyond DRS for diagnostics, other optical techniques have been successfully applied to evaluate the blood supply and oxygenation in pulmonary tissues. A rapid multispectral endoscopic imaging system was developed for the near real-time mapping of the blood volume fraction and oxygen saturation in the lung mucosa [66]. Laser Doppler flowmetry has been validated for monitoring microcirculatory blood flow in lung tissue in animal models [67]. More recently, advanced techniques like multiscale 3D imaging [68] and optical oxygen saturation imaging during ex vivo lung perfusion [69] have provided detailed quantitative assessments of the pulmonary vasculature and function.

In summary, while various optical techniques, including DRS, have been successfully applied to the study of lung tissues—mostly for oncological diagnostics or in animal/ex vivo models—this underscores the broader potential of optical methods in respiratory medicine. However, the specific application of DRS for the intraoperative quantitative assessment of tracheal wall blood supply and oxygenation to prevent anastomotic complications remains a novel contribution of the present work. The existence of the cited studies confirms the technical possibility, while our research addresses a distinct and unmet clinical need in tracheal surgery.

### 4.3. Statistics

Without StO_2_ measurements, the frequency of anastomosis failures during tracheal resection under the same other conditions was under 3% of cases. While with the use of the diffuse reflectance spectroscopy method, failure was observed in one patient. However, in this case, the occurrence of postoperative complications was caused by mechanical damage, which allows us to exclude the data of this patient from the sample. Thus, the failure rate using the developed technique is 0%. Accordingly, there is a tendency to decrease the number of cases of anastomosis failure, but a larger sample is needed to confirm the statistical significance of these differences.

The data samples for each intraoperative measurement stage are dependent, since the same parameter (StO_2_) was determined in certain areas but at different times during the surgical procedure. For pairwise comparison of the measurement results for each stage, the Wilcoxon test values were determined. The analysis shows statistically significant differences between the groups of data derived at stages 1 and 2 (W = 19) with a significance level of *p* = 0.01, as well as at stages 2 and 3 (W = 66) with a significance level of *p* = 0.05. According to the Wilcoxon test, there are no statistically significant differences between the measurement results at stages 1 and 3 (W = 81), which confirms the restoration of the degree of blood supply to the initial level after the anastomosis. The Friedman test was used to simultaneously compare all data groups. For comparison the measurement results at all three stages, it is equal to 13.4, which indicates the presence of statistically significant differences with a significance level of *p* = 0.01.

In summary, diffuse scattering fiber spectroscopy shows real potential as an additional tool for the intraoperative assessment of tracheal wall blood supply—especially in the adventitial approach—but more rigorous methodological development, expanded validation, and comparative studies with established perfusion monitoring methods are needed to move to clinical recommendations. The ongoing progress in optical methods for personalized medicine, as reviewed elsewhere [70], supports the feasibility and future development of such quantitative non-invasive diagnostic approaches.

## 5. Conclusions

The study demonstrates that the developed method based on diffuse scattering fiber spectroscopy allows the quantitative assessment of the parameters of blood supply to the tracheal wall layers under intraoperative conditions. The experimentally extracted StO_2_ values are consistent with the simulated scenarios, which increases the confidence in the used oxygenation extraction algorithm. The key practical conclusion is that the placement of the probe from the adventitia side provides higher sensitivity to changes in oxygenation of the deep layers (fibrocartilage complex), while access from the mucosa reflects mainly superficial microcirculation. The method has the advantages of non-contrast and simple hardware implementation; therefore, it can act as a targeted tool for assessing suspicious areas around the anastomosis. The presented technique allows influencing the outcome of the surgical procedure by reducing the risk of serious postoperative complications, including anastomotic failure. The method can be used during resection of malignant neo-plasms of the trachea. At the same time, the current results are from a pilot: they are limited by a small sample size, simplifications in modeling, and point measurements. To move to clinical recommendations, extended validation on a larger sample, parallel comparison with reference methods (ICG-FA, HSI, LSCI, PAI), and the development of standardized measurement and interpretation protocols are needed.

## Figures and Tables

**Table 1:** Simulation parameters for each layer.

Layers	Thickness of the Layer, mm	cm^−1^	500 nm	542 nm	556 nm	576 nm	586 nm
mucosa (solid structure)	0.35	μ_a_	2.9	3.6	3.3	3.5	3
		μ_s_	145	142	142	141	140
		g	0.71	0.73	0.74	0.75	0.76
submucosa (solid structure)	0.55	μ_a_	2.2	2.8	2.5	2.7	2
		μ_s_	143	140	140	139	139
		g	0.69	0.72	0.73	0.74	0.75
fibrocartilaginous membrane (solid structure) 1 mm	perichondrium 0.1	μ_a_ oxy	5.42	6.81	4.82	6.84	4.49
		μ_a_ deoxy	5.15	6.54	5.89	6.01	4.83
		μ_s_	127	120	120	120	119
		g	0.77	0.79	0.8	0.82	0.79
	0.8	μ_a_	2.6	4	3	3.9	3.1
		μ_s_	127	120	120	120	119
		g	0.77	0.79	0.8	0.82	0.79
	perichondrium 0.1	μ_a_ oxy	5.42	6.81	4.82	6.84	4.49
		μ_a_ deoxy	5.15	6.54	5.89	6.01	4.83
		μ_s_	127	120	120	120	119
		g	0.77	0.79	0.8	0.82	0.79
adventitia (solid structure)	0.1	μ_a_	2.1	3.5	2.8	3.5	3
		μ_s_	80	78	77	75	72
		g	0.85	0.86	0.87	0.88	0.88
Hb		μ_a_	112	285	185	297	142
HbO_2_		μ_a_	112	258	292	215	176

## Data Availability

The raw data supporting the conclusions of this article will be made available by the authors on request.

## Abbreviations

The following abbreviations are used in this manuscript:

StO_2_	Oxygen saturation
OCA	Optical coherence angiography
NIR	Near infrared
ICG-FA	Indocyanine green fluorescence angiography
HSI	Hyperspectral imaging
LSCI	Laser speckle contrast imaging
PAI	Photoacoustic imaging
DRS	Diffuse reflectance spectroscopy
LED	Light emitting diode
PC	Personal computer
Rd	Diffuse reflection
Td	Diffuse transmission
Hb	Deoxygenated hemoglobin
HbO_2_	Oxygenated hemoglobin
